# Relationship between the circulating N-terminal pro B-type natriuretic peptide and the risk of carotid artery plaque in different glucose metabolic states in patients with coronary heart disease: a CSCD-TCM ^*plus*^ study in China

**DOI:** 10.1186/s12933-023-02015-y

**Published:** 2023-11-02

**Authors:** Tong Yang, Hongmei Zheng, Guangwei Pan, Ruiying Guo, Fengmin Liu, Shengyuan Liu, Shuang Tao, Lin Li, Rongrong Yang, Chunquan Yu

**Affiliations:** 1 Tianjin University of Traditional Chinese Medicine, No. 10 Poyang Lake Road, Wet Zone, Tuanbo New City, Jinghai District, Tianjin, 301617 China; 2 Tianjin Medical University General Hospital, Tianjin, China

**Keywords:** Circulating N-terminal pro B-type natriuretic peptide, Carotid artery plaque, Glucose metabolic states, Coronary heart disease

## Abstract

**Objective:**

Circulating N-terminal pro B-type natriuretic peptide (NT-proBNP) is a marker for heart failure in patients with coronary heart disease (CHD) and associated with glycemic abnormalities. Studies on the association and diagnostic value of NT-proBNP in carotid plaques (CAP) in patients with CHD are limited.

**Methods:**

The relationships between NT-proBNP and the risk of CAP in different glucose metabolic states, sexes, and age categories were also examined using 5,093 patients diagnosed with CHD. The NT-proBNP tertiles were used to divide patients into three groups in which the NT-proBNP levels, blood glucose levels, the occurrence of CAP, and the number and nature of CAP were measured using normoglycemic (NG), prediabetes (Pre-DM), and diabetes mellitus (DM) glucose metabolic statuses. Logistic regression analyses were used to compare the relationship between NT-proBNP and the risk of CAP occurrence and the number and nature of CAP. The diagnostic value of NT-proBNP for CAP risk was measured using receiver operating characteristic (ROC) curves.

**Results:**

We found a 37% relative increase in the correlation between changes in NT-proBNP per standard deviation (SD) and the incidence of CAP. After adjusting for potential confounders, NT-proBNP at the T3 level was found to be associated with an increased CAP odds ratio (OR) when T1 was used as the reference. This relationship was also present in males, patients aged > 60 years, or both pre-DM and DM states. NT-proBNP was more likely to present as hypoechoic plaques at T1 and as mixed plaques at T3. We also measured the diagnostic accuracy of CAP for NT-proBNP in patients with CHD, with an AUC value of 0.627(95% CI 0.592–0.631), sensitivity of 50.7%, and specificity of 68.0%.

**Conclusion:**

An increase in NT-proBNP was significantly associated with the risk of CAP in patients with CHD, especially in males and patients aged > 60 years, and exhibited specific characteristics under different glucose metabolism states.

*Trial registration* The study was approved by the Ethics Committee of Tianjin University of Traditional Chinese Medicine (Approval number TJUTCM-EC20210007) and certified by the Chinese Clinical Trials Registry on April 4, 2022 (Registration number ChiCTR2200058296) and March 25, 2022 by ClinicalTrials.gov (registration number NCT05309343).

**Graphical Abstract:**

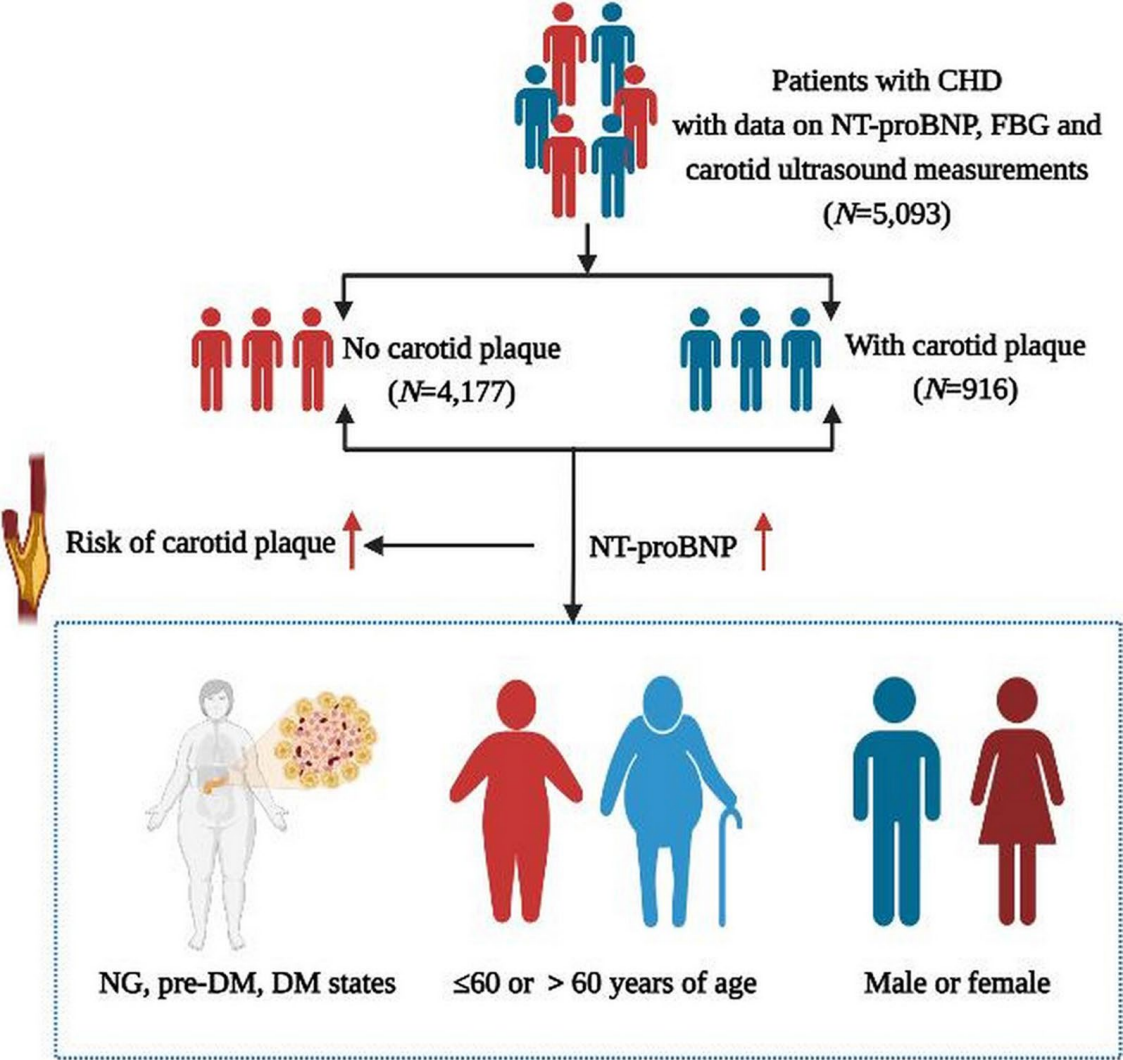

**Supplementary Information:**

The online version contains supplementary material available at 10.1186/s12933-023-02015-y.

## Background

Coronary heart disease (CHD) is a chronic noncommunicable disease whose increasing annual morbidity and mortality represent a major global public health burden [1]. While its pathogenesis is closely linked to the presence of endothelial dysfunction [2], diabetes mellitus (DM) is another important risk factor independent of the traditional risk factors of CHD, which include hypertension, hyperlipidemia, and smoking, and is associated with a 2–threefold increase in the incidence of CHD, myocardial infarction (MI), and CHD mortality [3]. In addition, CHD often occurs in combination with DM, possibly because the risk factors for both include abnormal inflammatory responses and abnormal lipid metabolism [4]. The early detection of atherosclerosis, especially in asymptomatic patients with CHD, is crucial to patient treatment and survival. Carotid artery plaque (CAP) is an atherosclerotic lesion thought to predict poor outcomes of CHD and can be used as a surrogate for atherosclerotic disease [5]. Previous studies have found that abnormalities in glucose metabolism are strongly associated with the risk of developing carotid artery plaque [6, 7].

Circulating N-terminal pro B-type natriuretic peptide (NT-proBNP) is a common diagnostic and prognostic marker for heart failure. It is an inactive amino acid fragment of the brain natriuretic peptide (BNP) that is released by cardiomyocytes in response to volume or pressure overload and promotes vasodilation and natriuretic effect [8]. NT-proBNP is a better indicator of diabetes mellitus than traditional risk factors, may better at differentiating the risk of death from cardiovascular disease (CVD) in DM patients [9], and be useful in monitoring diabetes-related micro- and macro-vascular complications [10, 11]. However, there are no current studies on the association between NT-proBNP and CAP risk in different glucose metabolism states, and the association between NT-proBNP and CAP in the CHD population is also unknown. Therefore, the aim of this study was to compare the correlation between NT-proBNP and the risk of CAP in a Chinese population with CHD and confirm the relationship between NT-proBNP and the development of CAP under different glucose metabolism conditions, age, and sex.

## Methods

### Subjects

**Fig. 1:**
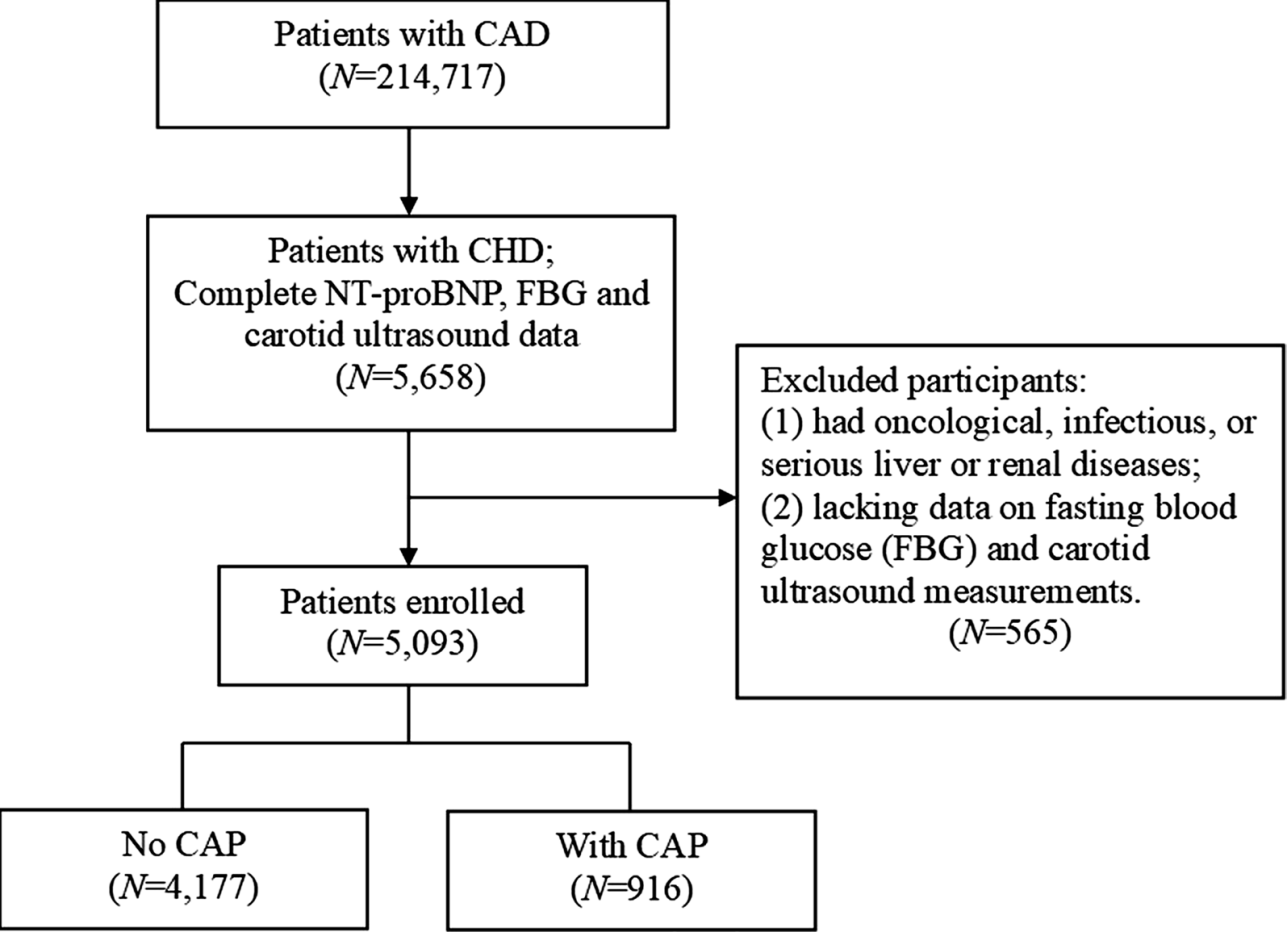
Flow chart of patient recruitment

We conducted a large multicenter cohort study named* Cohort Study on Treatment of Cardiovascular Diseases with Traditional Chinese Medicine (CSCD-TCM *^*plus*^*)*. During the study, we established a *CSCD-TCM *^*plus*^ database that included data on 214,717 individual hospital cases related to CAD from six hospitals in Tianjin, including the First Teaching Hospital of Tianjin University of Traditional Chinese Medicine, Tianjin Medical University General Hospital, Tianjin Hospital of ITCWM Nankai Hospital, Tianjin Chest Hospital, Tianjin Academy of Traditional Chinese Medicine Affiliated Hospital, and the Second Teaching Hospital of Tianjin University of Traditional Chinese Medicine, from January 1, 2014, to June 30, 2022. Patients with oncological, infectious, or serious liver or renal diseases, or those lacking data on fasting blood glucose (FBG) and carotid ultrasound measurements were excluded. Ultimately, 5,093 eligible subjects were included in the final analysis. A flowchart of patient recruitment is shown in Fig. 1.

### Data collection

The data for the analysis of this study obtained from the CSCD-TCM ^*plus*^ database included data that came from patient medical records: clinical history, anthropometric data, blood analysis, and medical imaging data. Anthropometric data, including blood pressure and personal information such as age, sex, smoking status, drinking status, previous percutaneous coronary intervention (PCI), coronary artery bypass grafting (CABG), MI, current antihypertensive treatment, and current anti-lipid treatment, were recorded. The left ventricular ejection fraction (LVEF) and New York Heart Association (NYHA) functional classifications were also recorded. Fasting venous blood samples were collected from all patients on the second day of hospitalization. Patients were admitted to the hospital for standardized procedure-based testing of a single random urine sample using an automated urine analyzer. Carotid artery ultrasonography examinations were performed by a certified professional technician using a diagnostic ultrasonography system. The common carotid artery, internal carotid artery, and carotid artery bifurcation were scanned and visualized using B-mode imaging. Carotid artery intima-media thickness (CIMT) was determined as the average IMT of the left and right common carotid arteries [12]. Professional physicians analyzed the color of the carotid artery based on Doppler ultrasound results and recorded the number of CAPs and ultrasonographic features. CAP cases were divided into single (n = 1) and multiple (n ≥ 2). The echogenic properties of CAP were categorized into hypoechogenic, isoechogenic, hyperechogenic, and mixed types. Strict quality control procedures were used to maintain consistency in the acquisition and analysis of the monitoring and test images. Inter-laboratory quality was assessed by licensed experimenters [13].

### Definitions

Smokers were defined as those who smoke at least 100 cigarettes in their lifetime [14]. Drinkers were defined as those who consumed alcohol at least 1 time per week [15]. Hypertension was defined as systolic blood pressure (SBP) ≥ 140 mmHg and/or diastolic blood pressure (DBP) ≥ 90 mmHg or current use of antihypertensive medication [16]. Diabetic status included normoglycemia (NG) (FBG < 5.6 mmol/L), prediabetes (Pre-DM) (5.6 ≤ FBG ≤ 6.9 mmol/L), and DM (FBG ≥ 7.0 mmol/L) [17]. Urinalysis showed qualitative protein results of + -, 1+, 2 +, 3 +, or 4+ . Proteinuria was considered present. Elevated serum cholesterol and/or triglyceride (TG) levels were reported as hyperlipidemia. Total cholesterol (TC) ≥ 5.2 mmol/L or TG ≥ 1.7 mmol/L or low-density lipoprotein cholesterol (LDL-C) ≥ 3.4 mmol/L were also reported [18]. Estimation of glomerular filtration rate (eGFR) was calculated using a formula previously described in Chinese patients with chronic kidney disease patients [19].

### Statistical analysis

The NT-proBNP terms include T1 (NT-proBNP < 56 ng/L), T2 (56 ≤ NT-proBNP ≤ 480 ng/L) and T3 (NT-proBNP > 480 ng/L). The Kolmogorov–Smirnov test was used to evaluate the normality of the data. Demographic differences between groups were assessed using the Kruskal–Wallis H test. Logistic regression models were estimated using odds ratios (ORs) and 95% confidence intervals (95% CIs) and used to examine the association between NT-proBNP and CAP. The collinearity of different models was tested using logistic regression. The diagnostic value of NT-proBNP measurements for predicting the risk of developing CAP was assessed using receiver operating characteristic curves. The missing values were imputed using multiple imputation methods.

Prior to the multifactor analyses, univariate analyses of CAP-related factors [20] were conducted to determine the inclusion of model confounders in the regression. Model 1 was unadjusted; Model 2 was adjusted for age and sex; and Model 3 was adjusted for age, sex, smoking, drinking, hypertension, diabetes, hyperlipidemia, EF%, proteinuria, previous MI, PCI or CABG, use of antihypertensives, and use of antilipidemic drugs. Statistical differences in the associations between the subgroups were analyzed using a multiplicative interaction approach. We also calculated the c-index and net reclassification improvement (NRI) to assess the accuracy of predicting CAP after including the factors used in Models 2 and 3 in Model 1.

Results with a two-sided *P* < 0.05 was considered statistically significant. All statistical analyses were performed using Statistical Package for the Social Sciences, version 26.0 (IBM Corp, New York, NY, USA).

## Results

### Subject characteristics

A total of 5093 participants were included in this study, of which 2490 (49.0%) were female and 2,597 (51.0%) were male, with a median age of 65 years; 3632 (71.3%) were over the age of 60 years. 1807 (35.5%), 1325 (26.0%), and 1961 (38.5%) were NG, pre-DM, and DM, respectively. The subjects were divided into three groups at the tertile level of the NT-proBNP index (e.g., T1, T2, and T3). When compared to the T1 group, the distribution of HbA1c, c-reactive protein (CRP), percentage of proteinuria, and NYHA class III and IV levels were higher in the T2 and T3 groups, while the TG, TC LDL-C, LVEF were lower in the T2 and T3 groups. The incidence of CAP was highest in the T3 group. The baseline characteristics of the participants according to NT-proBNP levels are shown in Table 1.

**Table 1:** Baseline clinical characteristics according to NT-proBNP

Characteristics	Total	T1 *N* = 1687	T2 *N* = 1710	T3 *N* = 1696	*P-*value
Age					< 0.001
Total	65(60,71)	62(56,67)	66(61,70)	67(61,71)	
≤ 60	1461(28.7)	700(41.5)	390(22.8)	371(21.9)	
> 60	3632(71.3)	987(58.5)	1320(77.2)	1325(78.1)	
Sex					< 0.001
Male	2597(51.0)	867(51.4)	775(45.2)	955(56.3)	
Female	2490(49.0)	820(48.6)	935(54.7)	741(43.7)	
SBP (mmHg)	140.0(130.0,160.0)	140.0(130.0,160.0)	143.0(130.0,160.0)	140.0(127.0,160.0)	0.061
DBP (mmHg)	81.0(80.0,90.0)	85.0(80.0,93.0)	80.0(80.0,90.0)	80.0(76.0,90.0)	< 0.001
Drinking (%)	1242(24.4)	459(27.2)	398(23.3)	385(22.7)	0.004
Smoking (%)	2105(41.3)	728(43.2)	687(40.2)	690(40.7)	0.170
Hypertension (%)	3915(76.9)	1336(79.2)	1372(80.2)	1207(71.2)	< 0.001
Hyperlipidemia (%)	902(17.7)	402(23.8)	324(18.9)	176(10.4)	< 0.001
HbA1c (%)	6.1(5.6,7.1)	6.0(5.5,6.9)	6.1(5.6,7.1)	6.3(5.6,7.5)	< 0.001
FBG (mmol/l)	6.2(5.2,8.3)	6.3(5.3,8.3)	6.2(5.2,8.1)	6.4(5.2,8.7)	0.019
CRP (mg/l)	3.5(2.0,7.4)	2.6(1.5,4.3)	2.8(1.8,4.9)	6.0(2.9,15.4)	< 0.001
eGFR (ml/min/1.73m^2^)	109.8(88.3,131.1)	110.1(91.8,129.0)	110.2(84.2,126.9)	108.5(83.0,130.2)	0.831
LVEF (%)	61.0(56.8,65.0)	63.0(60.0,66.0)	62.0(59.0,65.0)	58.0(51.0,62.0)	< 0.001
TG (mmol/l)	1.4(1.0,2.1)	1.5(1.1,2.1)	1.4(1.0,2.0)	1.2(0.9,1.7)	< 0.001
TC (mmol/l)	4.3(3.6,5.1)	4.5(3.8,5.2)	4.5(3.7,5.2)	4.0(3.3,4.9)	< 0.001
LDL-C (mmol/l)	2.8(2.2,3.5)	3.0(2.4,3.6)	2.9(2.3,3.6)	2.6(2.0,3.4)	< 0.001
HDL-C (mmol/l)	0.9(1.0,1.3)	1.0(0.9,1.3)	1.1(0.9,1.3)	1.0(0.8,1.2)	< 0.001
Proteinuria (%)	283(5.6)	25(1.5)	65(3.8)	193(11.4)	< 0.001
NYHA classification (%)					< 0.001
I	558(11.0)	288(17.1)	190(11.1)	80(4.7)	
II	3049(59.9)	1180(69.9)	1181(69.1)	688(40.6)	
III	1201(23.6)	197(11.7)	298(17.4)	706(41.6)	
IV	285(5.6)	22(1.3)	41(2.4)	222(13.1)	
Previous PCI (%)	462(9.1)	129(7.6)	159(9.3)	174(10.3)	0.028
Previous CABG (%)	123(2.4)	20(1.2)	38(2.2)	65(3.8)	< 0.001
Previous MI (%)	616(12.1)	112(6.6)	173(10.1)	331(19.5)	< 0.001
Glucose regulation state (%)					0.021
NG	1807(35.5)	583(34.6)	647(37.8)	577(34.0)	
Pre-DM	1325(26.0)	472(28.0)	427(25.0)	426(25.1)	
DM	1961(38.5)	632(37.5)	636(37.2)	693(40.9)	
Current antihypertensive medication (%)	3126(61.4)	1012(60.0)	1069(62.5)	1045(61.6)	0.309
Current antilipidemic medication (%)	3808(74.8)	1282(76.0)	1305(76.3)	1221(72.0)	0.005
CIMT (cm)	0.9(0.8,1.1)	0.9(0.8,1.0)	0.9(0.8,1.1)	1.0(0.9,1.1)	< 0.001
Carotid artery plaque (%)	4177(82.0)	1270(75.3)	1408(82.3)	1499(88.4)	< 0.001
Number of carotid artery plaque (%)					< 0.001
0	916(18.0)	417(24.7)	302(17.7)	197(11.6)	
1	399(7.8)	160(9.5)	146(8.5)	93(5.5)	
≥ 2	3778(74.2)	1110(65.8)	1262(73.8)	1406(82.9)	
Carotid artery plaque echo property (%)					< 0.001
Hypoechoic plaque	192(3.8)	86(5.1)	49(2.9)	57(3.4)	
Isoechoic plaque	379(7.4)	148(8.8)	128(7.5)	103(6.1)	
Hyperechoic plaque	2413(47.4)	691(41.0)	809(47.3)	913(53.8)	
Mixture plaque	1200(23.6)	348(20.6)	423(24.7)	429(25.3)	

Data are presented as median (interquartile) or number (proportion, %)

SBP, systolic blood pressure; DBP, diastolic blood pressure; HbA1c, glycated hemoglobin; FBG, fasting blood glucose; CRP, C-reactive protein; eGFR, estimated glomerular filtration rate; LVEF, Left ventricular ejection fraction; TG, triglycerides; TC, total cholesterol; LDL-C, low-density lipoprotein cholesterol; HDL-C, high-density lipoprotein cholesterol; NYHA, New York Heart Association; PCI, percutaneous coronary intervention; CABG, coronary artery bypass grafting, MI, myocardial infarction; NG, normoglycemic; Pre-DM, pre-diabetes; DM, diabetes; CIMT, carotid intima-media thickness

### Association between the NT-proBNP and the risk of carotid artery plaques

Our multivariate logistic regression analyses showed that there was a 37% relative increase in the correlation between changes in NT-proBNP per SD and the incidence of CAP (OR: 1.37; 95% CI 1.01–1.85; Table 2). In model 3, which adjusted for potential confounders, NT-proBNP at the T3 level was associated with an increased CAP OR (OR: 1.67; 95% CI 1.26–2.22) when T1 was used as the reference. After correcting for potential confounders, the risk of CAP development was significantly associated with NT-proBNP at the T3 level in males, or in patients > 60 years of age when T1 was used as a reference (Tables 3 and 4).

**Table 2:** Association between the NT-proBNP and the risk of carotid artery plaque

Variables	Carotid artery plaques					
	OR (95% CI)^a^	*P-*value	OR (95% CI)^b^	*P-*value	OR (95% CI)^c^	*P-*value
NT-proBNP						
Continuous (per 1 SD increase)	1.76(1.42–2.17)	< 0.001	1.38(1.14–1.68)	0.001	1.37(1.01–1.85)	0.043
< 56	Reference		Reference		Reference	
[56–480]	1.53(1.30–1.81)	< 0.001	1.10(0.92–1.32)	0.297	1.03(0.83–1.29)	0.775
> 480	2.50(2.08–3.01)	< 0.001	1.58(1.29–1.92)	< 0.001	1.67(1.26–2.22)	< 0.001
* P*_trend_	< 0.001		< 0.001		< 0.001	

^a^Model 1: unadjusted

^b^Model 2: adjusted for age, sex

^c^Model 3: adjusted for age, sex, smoking, drinking, hypertension, diabetes, hyperlipidemia, LVEF, proteinuria, previous MI, PCI or CABG, use of antihypertensives, and use of antilipidemic

**Table 3:** Association between the NT-proBNP and the risk of carotid artery plaque according to sex

Variables	Carotid artery plaques					
	OR (95% CI)^a^	*P-*value	OR (95% CI)^b^	*P-*value	OR (95% CI)^c^	*P-*value
NT-proBNP						
Males						
Continuous (per 1 SD increase)	1.26(1.03–1.55)	0.023	1.10(0.92–1.31)	0.316	1.27(0.88–1.82)	0.203
< 56	Reference		Reference		Reference	
[56–480]	1.48(1.13–1.94)	0.004	1.11(0.83–1.47)	0.488	1.07(0.76–1.51)	0.695
> 480	2.12(1.62–2.80)	< 0.001	1.49(1.12–1.99)	0.007	2.12(1.37–3.30)	0.001
* P*_trend_	< 0.001		0.007		< 0.001	
Females						
Continuous (per 1 SD increase)	3.04(1.96–4.72)	< 0.001	1.12(1.11–1.14)	< 0.001	1.52(0.93–2.47)	0.095
< 56	Reference		Reference		Reference	
[56–480]	1.68(1.25–2.08)	< 0.001	1.08(0.86–1.37)	0.508	0.98(0.73–1.31)	0.879
> 480	2.76(2.14–3.57)	< 0.001	1.62(1.23–2.14)	0.001	1.35(0.93–1.97)	0.118
* P*_trend_	< 0.001		< 0.001		0.001	

^a^Model 1: unadjusted

^b^Model 2: adjusted for age

^c^Model 3: adjusted for age, smoking, drinking, hypertension, diabetes, hyperlipidemia, LVEF, proteinuria, previous MI, PCI or CABG, use of antihypertensives, and use of antilipidemic

**Table 4:** Association between the NT-proBNP and the risk of carotid artery plaque according to age

Variables	Carotid artery plaques					
	OR (95% CI)^a^	*P-*value	OR (95% CI)^b^	*P-*value	OR (95% CI)^c^	*P-*value
NT-proBNP						
≤ 60						
Continuous (per 1 SD increase)	1.41(1.11–1.80)	0.005	1.41(1.10–1.81)	0.006	1.03(0.74–1.42)	0.877
< 56	Reference		Reference		Reference	
[56–480]	1.36(1.04–1.76)	0.023	1.32(1.01–1.72)	0.042	1.16(0.84–1.62)	0.372
> 480	2.01(1.51–2.67)	< 0.001	1.87(1.40–2.49)	< 0.001	1.46(0.95–2.25)	0.082
* P*_trend_	< 0.001		< 0.001		0.112	
> 60						
Continuous (per 1 SD increase)	2.11(1.50–2.98)	< 0.001	1.53(1.12–2.09)	0.008	1.74(1.17–2.60)	0.006
< 56	Reference		Reference		Reference	
[56–480]	1.12(0.89–1.42)	0.329	1.18(0.93.1.50)	0.167	1.09(0.81.1.46)	0.575
> 480	1.97(1.51–2.56)	< 0.001	1.90(1.46–2.48)	< 0.001	2.08(1.43–3.01)	< 0.001
* P*_trend_	< 0.001		< 0.001		< 0.001	

^a^Model 1: unadjusted

^b^Model 2: adjusted for sex

^c^Model 3: adjusted for sex, smoking, drinking, hypertension, diabetes, hyperlipidemia, LVEF, proteinuria, previous MI, PCI or CABG, use of antihypertensives, and use of antilipidemic

### Association between the NT-proBNP and the risk of carotid artery plaques according to glucose regulation state

In the NG group, the NT-proBNP per SD change was correlated with CAP occurrence (OR: 2.50; 95% CI 1.17–5.39) after multivariate adjustment. Using T1 as a reference, T3 was significantly associated with an increased risk of CAP in both the pre-DM (OR: 1.94; 95% CI 1.10–3.43) and DM (OR: 1.85; 95% CI 1.13–3.01) states (Table 5). After adjusting for confounders, T3 was associated with the risk of developing CAP in men with pre-DM state (OR: 3.32; 95% CI 1.27–8.69) when we used T1 as a reference (Table 6). After adjusting for possible confounders, there had a significantly increased risk of developing CAP with T3 in patients with CHD who were > 60 years of age in the both of pre-DM (OR: 3.44; 95% CI 1.53–7.76) and DM (OR: 2.32; 95% CI 1.15–4.65) state when we used T1 as a reference (Table 7). Together, these results indicated that elevated NT-proBNP correlates with CHD patients with abnormal glucose metabolism, and this correlation is present in both men and the elderly.

**Table 5:** Association between the NT-proBNP and the risk of carotid artery plaques according to glucose regulation state

Variables	Carotid artery plaques					
	OR (95% CI)^a^	*P-*value	OR (95% CI)^b^	*P-*value	OR (95% CI)^c^	*P-*value
Normal glucose regulation						
Continuous (per 1 SD increase)	2.99(1.91–4.68)	< 0.001	2.98(2.29–3.87)	< 0.001	2.50(1.17–5.39)	0.018
< 56	Reference		Reference		Reference	
[56–480]	1.39(1.07–1.79)	0.012	1.00(0.75–1.32)	0.971	0.87(0.61–1.23)	0.437
> 480	2.45(1.83–3.29)	< 0.001	1.43(1.03–1.97)	0.031	1.38(0.88–2.15)	0.161
* P*_trend_	< 0.001		0.015		0.002	
Prediabetes						
Continuous (per 1 SD increase)	1.25(0.98–1.60)	0.070	1.02(1.83–1.25)	0.875	1.14(0.80–1.62)	0.467
< 56	Reference		Reference		Reference	
[56–480]	1.85(1.34–2.57)	< 0.001	1.26(0.88–1.79)	0.205	1.27(0.83–1.95)	0.275
> 480	2.71(1.90–3.85)	< 0.001	1.48(1.10–2.20)	0.045	1.94(1.10–3.43)	0.023
* P*_trend_	< 0.001		0.089		0.003	
Diabetes						
Continuous (per 1 SD increase)	1.63(1.13–2.35)	0.009	1.40(0.99–1.98)	0.057	1.32(0.81–2.14)	0.266
< 56	Reference		Reference		Reference	
[56–480]	1.551(1.14–2.12)	0.005	1.16(0.84–1.60)	0.376	1.07(0.72–1.58)	0.743
> 480	2.34(1.68–3.25)	< 0.001	1.69(1.20–2.39)	0.003	1.85(1.13–3.01)	0.014
* P*_trend_	< 0.001		0.003		0.010	

^a^Model 1: unadjusted

^b^Model 2: adjusted for age, sex

^c^Model 3: adjusted for age, sex, smoking, drinking, hypertension, hyperlipidemia, LVEF, proteinuria, previous MI, PCI or CABG, use of antihypertensives, and use of antilipidemic

**Table 6:** Association between the NT-proBNP and the risk of carotid artery plaques according to different glucose regulation state and sex

Variables	Carotid artery plaques					
	OR (95% CI)^a^	*P-*value	OR (95% CI)^b^	*P-*value	OR (95% CI)^c^	*P-*value
Male						
Normal glucose regulation						
Continuous (per 1 SD increase)	1.99(1.22–3.24)	0.006	1.74(1.08–2.80)	0.023	1.56(0.78–3.15)	0.212
< 56	Reference		Reference		Reference	
[56–480]	1.37(0.90–2.08)	0.144	0.93(0.59–1.47)	0.742	1.05(0.61–1.81)	0.862
> 480	2.06(1.33–3.21)	0.001	1.35(0.82–2.20)	0.224	1.11(0.60–2.04)	0.737
* P*_trend_	0.004		0.131		0.767	
Prediabetes						
Continuous (per 1 SD increase)	0.98(0.8–1.21)	0.864	0.83(0.67–1.03)	0.090	0.95(0.63–1.41)	0.792
< 56	Reference		Reference		Reference	
[56–480]	1.93(1.13–3.31)	0.017	1.26(0.71–2.23)	0.430	1.35(0.68–2.71)	0.392
> 480	2.49(2.47–3.21)	0.001	1.47(0.84–2.58)	0.182	3.32(1.27–8.69)	0.015
* P*_trend_	0.007		0.255		0.020	
Diabetes						
Continuous (per 1 SD increase)	1.26(0.86–1.83)	0.237	1.16(0.82–1.64)	0.409	1.16(0.68–1.98)	0.588
< 56	Reference		Reference		Reference	
[56–480]	1.41(0.88–2.24)	0.153	1.22(0.76–2.00)	0.421	1.10(0.62–1.95)	0.734
> 480	1.98(2.24–3.16)	0.004	1.65(1.02–2.67)	0.043	2.05(1.99–4.28)	0.054
* P*_trend_	0.012		0.061		0.050	
Female						
Normal glucose regulation						
Continuous (per 1 SD increase)	4.429(1.92–9.96)	< 0.001	2.60(1.29–4.27)	0.008	1.68(0.57–4.93)	0.343
< 56	Reference		Reference		Reference	
[56–480]	1.49(1.07–2.07)	0.018	1.04(0.73–1.49)	0.816	0.80(0.50–1.26)	0.336
> 480	2.64(1.77–3.94)	< 0.001	1.50(0.97–2.32)	0.068	1.03(0.57–1.88)	0.925
* P*_trend_	< 0.001		0.053		0.621	
Prediabetes						
Continuous (per 1 SD increase)	2.01(1.04–3.89)	0.038	1.79(0.98–3.25)	0.056	1.46(0.73–2.92)	0.288
< 56	Reference		Reference		Reference	
[56–480]	1.92(1.27–2.90)	0.002	1.25(0.80–1.97)	0.335	1.23(0.70–2.14)	0.474
> 480	2.62(1.61–4.26)	< 0.001	1.51(0.87–2.56)	0.130	1.48(0.71–3.07)	0.293
*P*_trend_	0.002		0.197		0.366	
Diabetes						
Continuous (per 1 SD increase)	2.71(1.29–5.71)	0.009	2.00(1.03–3.90)	0.042	1.68(0.63–4.45)	0.297
< 56	Reference		Reference		Reference	
[56–480]	1.79(1.19–2.70)	0.005	1.01(0.64–1.58)	0.979	0.95(0.55–1.66)	0.859
> 480	2.76(1.74–4.38)	< 0.001	1.62(0.99–2.66)	0.056	1.60(0.81–3.15)	0.175
* P*_trend_	< 0.001		0.033		0.109	

^a^Model 1: unadjusted

^b^Model 2: adjusted for age

^c^Model 3: adjusted for age, smoking, drinking, hypertension, diabetes, hyperlipidemia, LVEF, proteinuria, previous MI, PCI or CABG, use of antihypertensives, and use of antilipidemic

**Table 7:** Association between the NT-proBNP and the risk of carotid artery plaques according to different glucose regulation state and age

Variables	Carotid artery plaques					
	OR (95% CI)^a^	*P-*value	OR (95% CI)^b^	*P-*value	OR (95% CI)^c^	*P-*value
≤ 60						
Normal glucose regulation						
Continuous (per 1 SD increase)	1.42(1.08–1.86)	0.012	1.34(1.03–1.75)	0.030	3.00(0.91–9.90)	0.072
< 56	Reference		Reference		Reference	
[56–480]	1.11(0.74–1.65)	0.621	1.08(0.72–1.62)	0.727	0.84(0.50–1.40)	0.493
> 480	1.76(1.10–2.83)	0.020	1.54(0.95–2.51)	0.082	1.68(0.87–3.50)	0.163
* P*_trend_	0.021		0.082		0.097	
Prediabetes						
Continuous (per 1 SD increase)	0.95(0.77–1.17)	0.637	0.94(0.77–1.16)	0.581	0.75(0.50–1.12)	0.162
< 56	Reference		Reference		Reference	
[56–480]	1.71(0.98–2.96)	0.058	1.81(1.02–3.20)	0.043	1.57(1.78–3.17)	0.206
> 480	1.79(1.04–3.11)	0.037	1.62(0.92–2.86)	0.095	0.89(0.36–2.21)	0.807
* P*_trend_	0.093		0.224		0.594	
Diabetes						
Continuous (per 1 SD increase)	3.41(1.30–8.93)	0.013	3.27(1.26–8.53)	0.015	1.51(0.55–4.15)	0.420
< 56	Reference		Reference		Reference	
[56–480]	1.66(1.02–2.68)	0.041	1.55(0.95–2.53)	0.078	1.50(0.82–2.76)	0.189
> 480	2.23(1.38–3.60)	0.001	2.18(1.34–3.54)	0.002	1.47(0.73–2.94)	0.282
* P*_trend_	0.005		0.005		0.438	
> 60						
Normal glucose regulation						
Continuous (per 1 SD increase)	2.71(1.79–7.74)	< 0.001	3.12(1.51–6.44)	0.002	1.94(0.76–4.96)	0.166
< 56	Reference		Reference		Reference	
[56–480]	1.16(0.80–1.67)	0.432	1.23(0.85–1.78)	0.280	1.05(0.66–1.68)	0.832
> 480	1.94(1.30–2.90)	0.001	1.85(1.23–2.78)	0.003	1.43(0.82–2.47)	0.208
* P*_trend_	0.001		0.005		0.179	
Prediabetes						
Continuous (per 1 SD increase)	1.61(0.97–2.68)	0.066	1.45(0.91–2.30)	0.121	7.06(1.95–25.81)	0.002
< 56	Reference		Reference		Reference	
[56–480]	1.24(0.80–1.93)	0.342	1.23(0.79–1.93)	0.359	1.24(0.72–2.15)	0.443
> 480	2.33(1.40–3.87)	0.001	2.08(1.25–3.48)	0.005	3.44(1.53–7.76)	0.002
*P*_trend_	0.001		0.007		0.003	
Diabetes						
Continuous (per 1 SD increase)	1.15(0.76–1.75)	0.501	1.18(0.86–1.63)	0.304	1.27(0.74–2.17)	0.383
< 56	Reference		Reference		Reference	
[56–480]	1.01(0.65–1.56)	0.979	1.05(0.67–1.63)	0.829	0.94(0.56–1.61)	0.824
> 480	1.74(1.07–2.81)	0.025	1.75(1.08–2.83)	0.023	2.32(1.15–4.65)	0.018
* P*_trend_	0.009		0.012		0.005	

^a^Model 1: unadjusted

^b^Model 2: adjusted for sex

^c^Model 3: adjusted for sex, smoking, drinking, hypertension, diabetes, hyperlipidemia, LVEF, proteinuria, previous MI, PCI or CABG, use of antihypertensives, and use of antilipidemic

### Diagnostic value of NT-proBNP in the risk of carotid artery plaques

**Fig. 2:**
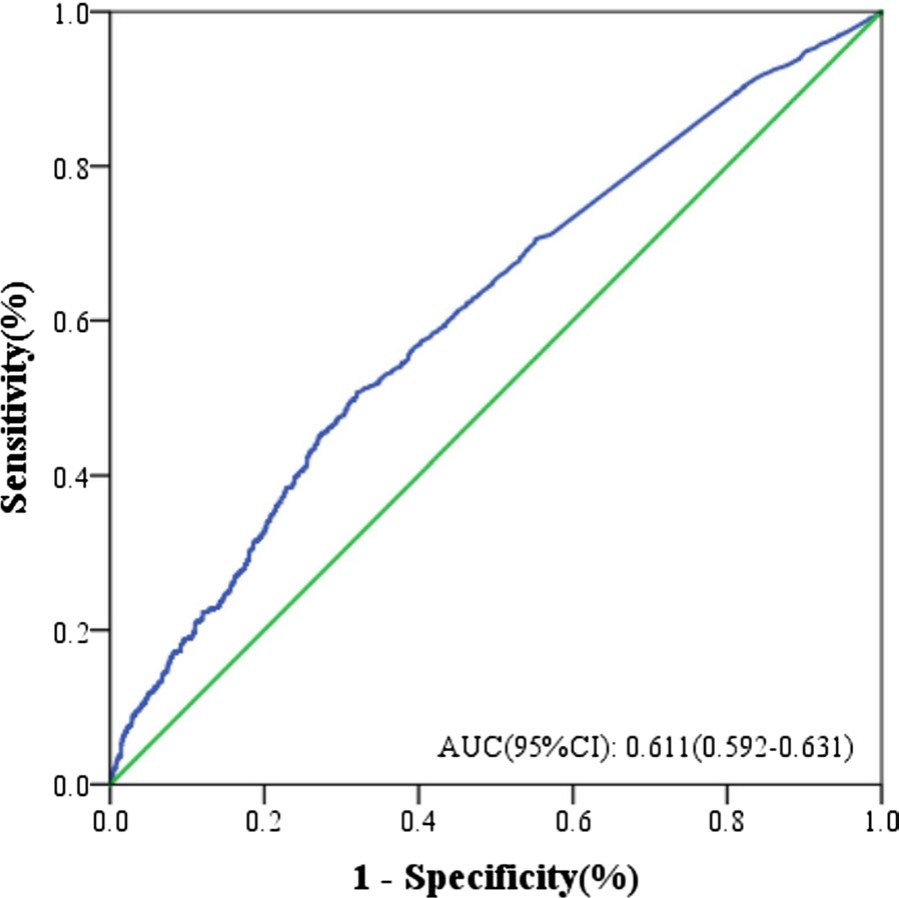
Diagnostic value of NT-proBNP for the risk of carotid artery plaques in patients with CHD

We measured the diagnostic accuracy of CAP for NT-proBNP levels in patients with CHD. The critical values were 182 ng/L, an AUC value of 0.627 (95% CI 0.592–0.631), sensitivity of 50.7%, and specificity of 68.0% (Fig. 2). These results showed that NT-proBNP has some validity in diagnosing the risk of CAP occurrence.

### Supplementary analysis

Further investigations of this study were performed using the electronic supplementary materials (ESM). Before performing the multifactorial analyses, we conducted univariate analyses related to CAP (Additional file 1: Table S1). Statistical differences in the associations among subgroups were analyzed using multiplicative interactions, which showed that the association between NT-proBNP and CAP was not statistically different at the level diabetes or age but was statistically different at the level of sex (Additional file 1: Table S2). We also calculated the c-index and NRI to assess the accuracy of predicting CAP after incorporating the factors from Models 2 and 3, which showed a significant increase in the c-index for these models when compared with Model 1, as well as an increase in the NRI after the model changed (Additional file 1: Table S3). When T1 was used as the reference, NT-proBNP at the T3 level was associated with the occurrence of ≥ 2 CAP cases (Additional file 1: Table S4), and was more likely to appear as hypoechoic plaques at the T1 level and as a mixture of plaques at the T3 level (Additional file 1: Table S5).

## Discussion

This study demonstrated the relationship between NT-proBNP and CAP in Chinese patients with CHD in different glucose metabolic states. This association was evaluated according to sex, age, and glucose metabolism. The NT-proBNP level was also found to have diagnostic significance for CAP.

Myocardial NT-proBNP synthesis and release are part of a counter-regulatory response to increased myocardial wall stress, sympathetic nerve tension, and vasoconstriction, acting in different systems and affecting various biological processes [21]. NT-proBNP and its receptors not only coordinate cardiovascular homeostasis and health by regulating blood pressure, blood volume, and sodium balance but are also involved in glucose and lipid metabolism in adipose and muscle tissues [22]. NT-proBNP also makes an additional contribution to the prediction of long-term CHD risk compared with conventional risk factors, including smoking, alcohol consumption, and hypertension [23], but is strongly associated with the risk of DM [24]. In addition, NT-proBNP levels are significantly associated with adverse cardiovascular events in patients with chronic CHD in the pre-diabetic state [25]. In the present study, we found that when NT-proBNP was used as a categorical variable, increased levels were associated with the risk of CAP, and it also had some validity as a diagnostic marker of CHD occurrence, which may be related to the close association between natriuretic peptides (NPs) and endothelial dysfunction. The biological action of the NPs is mediated through the guanylate cyclase (GC) receptor, which is present in many tissues, including the vascular endothelium and smooth muscle [26]. NPs and nitric oxide (NO) are important factors that exert synergistic vascular and cardiac actions. Increased cyclic guanosine monophosphate (cGMP) induced by NPs or NO is involved in vasorelaxation, inhibition of platelet aggregation and adhesion. The induction of NO synthase can lead to higher levels of NO, which is a potent vasodilator and marker of endothelial function [27]. Another possible mechanism is that NPs may inhibit the production and secretion of endothelin-1 (ET-1), which is also a potent vasoconstrictor [28].

NT-proBNP level is a good predictor of CHD outcomes in certain patient groups. Cosson et al. [29] found that in patients with diabetes, NT-proBNP was a useful biomarker for CHD diagnosis. Given the increasing prevalence of atrial disease and DM, and their increased cardiovascular burden [30, 31], investigating patients with impaired glucose metabolism and exploring more valuable prognostic biomarkers is of research and clinical interest. To the best of our knowledge, this is an examination of the relationship between NT-proBNP and the risk of incidence of CAP in different glucose metabolism states and found that the incidence of CAP increased with each standard deviation change in NT-proBNP, which existed only in the NG state. When divided into tertiles, it was significant in both the pre-DM and DM states when the < 56 ng/L group was used as the reference. The 480 ng/L group had a significantly higher risk of developing CAP, possibly from the potential presence of elevated NT-proBNP as a protective factor against DM, which is consistent with previous studies that showed NT-proBNP concentration was negatively associated with the future risk of DM in a population-based cohort study [10]. Similar observations were reported in the Community Atherosclerosis Risk Study, in which individuals with low NT-proBNP levels had a significantly higher risk of diabetes [32]. Therefore, when low NT-proBNP levels were used as a reference, high NT-proBNP and blood glucose levels acted together to induce inflammation, oxidative stress, and metabolic changes. This inflammation led to vascular endothelial damage and increased plaque incidence.

The concentration of NT-proBNP was reported to gradually increase with age, and is significantly higher in women than in men [33]. Given that the interpretation of NT-proBNP must account for specific differences in age and sex, we compared the association of NT-proBNP with CAP, as well as under different glucose metabolisms. When accounting for sex and age, we found that this association was significant in the pre-DM state in men and the pre-DM and DM states in patients aged > 60 years. This result may be attributed to differences in endothelin and angiotensin-converting enzyme activities due to sex-dependent hormonal status and structural changes in the heart due to advancing age.

The nature and quantity of plaques determine the process of rupture or erosion, which can cause adverse cardiovascular events in a short period of time [34, 35]. Therefore, detecting changes in NT-proBNP levels and atherosclerotic plaque characteristics in patients with CHD may have clinical significance. In this study, a high NT-proBNP level was associated with the formation of multiple CAPs, and the corresponding plaques were more likely to be mixed, which is consistent with previous reported of high NT-proBNP level corresponding to a large number of calcified plaques [36]. The presence of multiple plaques indicates severe luminal stenosis and calcified plaques often coexist with more severe luminal stenosis in the middle and late stages of the disease [37, 38] as well as being associated with inflammatory processes. Once plaque rupture or erosion occurs, the extent of myocardial infarction may be more severe than in early disease with noncalcified plaques. Therefore, factors such as NT-proBNP that can predict and diagnose the risk of CAP should be seriously considered by researchers and clinicians.

## Strengths and limitations

To our knowledge, this is the largest population-based study on the association between NT-proBNP and CAP in patients with CHD. This study validated this association in groups with different glucose metabolism, age, and sex to examine the characteristics of different populations. The analyses included possible confounders to exclude interference in the results. In addition, we investigated the diagnostic value of NT-proBNP for CAP, which may be useful for expanding the clinical application of this marker for monitoring and diagnosing atherosclerosis. However, this study had some limitations. As an observational study, it was not suitable for investigating the causal relationship between NT-proBNP and CAP. Additionally, factors such as glucose-lowering medications and body mass index (BMI) that are currently used as important confounders were not included in the regression model due to missing data. Finally, because this was a multicenter study, there may have been some unavoidable between-center bias that could have affected our results.

## Conclusions

NT-proBNP is closely related to the risk of CAP formation in different glucose metabolic states in patients with CHD. This relationship was especially significant in males and those > 60 years old, in men with pre-DM, and in patients > 60 years old with pre-DM and DM status. High NT-proBNP levels were strongly associated with high echogenicity and multiple CAPs. In patients with CHD and abnormal glucose metabolism, clinicians should monitor for elevated NT-proBNP levels and consider its association with CAP.

### Supplementary Information

**Additional file 1:** **Table S1.** Univariate analysis related to CAP. **Table S2.** Statistical differences for associations across subgroups. **Table S3.** Risk prediction accuracy of NT-proBNP for the tested outcome. **Table S4.** Association between the NT-proBNP and the number of carotid artery plaque. **Table S5.** Association between the NT-proBNP and the echogenicity of carotid artery plaque.

## Data Availability

The datasets used and/or analyzed in the current study are available from the corresponding author upon reasonable request.

## Abbreviations

CHD: Coronary heart disease
DM: Diabetes mellitus
MI: Myocardial infarction
CAP: Carotid artery plaque
NT-proBNP: Circulating N-terminal pro B-type natriuretic peptide
BNP: Brain natriuretic peptide
CAD: Cardiovascular disease
PCI: Percutaneous coronary intervention
CABG: Coronary artery bypass grafting
LVEF: Left ventricular ejection fraction
NYHA: New York Heart Association
CIMT: Carotid intima-media thickness
SBP: Systolic blood pressure
DBP: Diastolic blood pressure
NG: Normoglycemic
Pre-DM: Pre-diabetes
TG: Triglycerides
TC: Total cholesterol
LDL-C: Low-density lipoprotein cholesterol
ORs: Odds ratios
CI: Confidence intervals
CRP: C-reactive protein
HDL-C: High-density lipoprotein cholesterol
NPs: Natriuretic peptides
GC: Guanylate cyclase
cGMP: Cyclic guanosine monophosphate
NO: Nitric oxide
ET-1: Endothelin-1
BMI: Body mass index
