# Supplementary material for: Relationship between the circulating N-terminal pro B-type natriuretic peptide and the risk of carotid artery plaque in different glucose metabolic states in patients with coronary heart disease: a CSCD-TCM plus study in China
Source: Cardiovasc Diabetol. 2023 Nov 2;22:299. doi: 10.1186/s12933-023-02015-y (PMC10623780; doi:10.1186/s12933-023-02015-y)
Supplement: Supplementary file 1 — Additional file 1: Table S1. Univariate analysis related to CAP. Table S2. Statistical differences for associations across subgroups. Table S3. Risk prediction accuracy of NT-proBNP for the tested outcome. Table S4. Association between the NT-proBNP and the number of carotid artery plaque. Table S5. Association between the NT-proBNP and the echogenicity of carotid artery plaque. [file 12933_2023_2015_MOESM1_ESM.docx]

**Table S1 Univariate analysis related to CAP**

| Variables | OR (95% CI) | *P* value |
| --- | --- | --- |
| Age | 1.09(1.08-1.10) | <0.001 |
| Sex | | |
| Female | Reference |  |
| Male | 1.79(1.55-2.07) | <0.001 |
| SBP | 1.01(1.00-1.01) | <0.001 |
| DBP | 0.99(0.99-1.00) | 0.004 |
| Drinking | | |
| No | Reference |  |
| Yes | 1.50(1.26-1.80) | <0.001 |
| Smoking | | |
| No | Reference |  |
| Yes | 1.56(1.34-1.82) | <0.001 |
| Hypertension | | |
| No | Reference |  |
| Yes | 1.78(1.52-2.09) | <0.001 |
| Hyperlipidemia | | |
| No | Reference |  |
| Yes | 0.89(0.74-1.06) | 0.188 |
| HbA1c | 1.31(1.23-1.39) | <0.001 |
| FBG | 1.09(1.06-1.12) | <0.001 |
| eGFR | 0.97(0.91-1.05) | 0.461 |
| LVEF | 0.97(0.96-0.99) | <0.001 |
| TG | 0.94(0.88-1.00) | 0.065 |
| TC | 0.95(0.88-1.02) | 0.126 |
| LDL-C | 1.02(0.93-1.12) | 0.627 |
| HDL-C | 0.62(0.47-0.82) | 0.001 |
| Proteinuria | | |
| No | Reference |  |
| Yes | 1.83(1.26-2.68) | 0.002 |
| **Previous PCI** | | |
| No | Reference |  |
| Yes | 2.20(2.60-3.03) | <0.001 |
| **Previous CABG** | | |
| No | Reference |  |
| Yes | 5.3(2.2-13.0) | <0.001 |
| **Previous MI** | | |
| No | Reference |  |
| Yes | 2.15(1.63-2.83) | <0.001 |
| Current antihypertensive medication | | |
| No | Reference |  |
| Yes | 1.54(1.33-1.78) | <0.001 |
| Current antilipidemic medication | | |
| No | Reference |  |
| Yes | 1.61(1.38-1.88) | <0.001 |

**Table S2 Statistical differences for associations across subgroups**

| Variables | ***β*** | **SE** | ***P*-value** | **OR(95% CI)** |
| --- | --- | --- | --- | --- |
| Model ^3^ | | | | |
| NT-proBNP | 0.598 | 0.131 | <0.001 | 1.819(1.406-2.353) |
| DM | 0.516 | 0.085 | <0.001 | 1.675(1.419-1.977) |
| NT-proBNP × DM | -0.129 | 0.221 | 0.561 | -0.879(0.570-1.357) |
| Model ^3^ | | | | |
| NT-proBNP | 1.064 | 0.215 | <0.001 | 2.897(1.900-4.419) |
| Male | 0.440 | 0.084 | <0.001 | 1.553(1.316-1.833) |
| NT-proBNP × Male | -0.819 | 0.241 | 0.001 | 0.441(0.275-0.707) |
| Model ^3^ | | | | |
| NT-proBNP | 0.404 | 0.145 | 0.005 | 1.497(1.127-1.989) |
| >60 | 1.268 | 0.080 | <0.001 | 3.552(3.035-4.159) |
| NT-proBNP × >60 | 0.122 | 0.203 | 0.549 | 1.130(0.758-1.683) |

Model 3: adjusted for age, sex, smoking, drinking, hypertension, diabetes, hyperlipidemia, LVEF, proteinuria, previous MI, PCI or CABG, use of antihypertensives, and use of antilipidemic

**Table S3 Risk prediction accuracy of NT-proBNP for the tested outcome**

| Model | C-statistics | 95%CI | *Z* | *P* value | NRI |
| --- | --- | --- | --- | --- | --- |
| Model ^1^ Reference | 0.611 | 0.592-0.631 | - | - | - |
| Model ^2^ | 0.747 | 0.729-0.765 | 8.843 | ＜0.001 | 0.192 |
| Model ^3^ | 0.749 | 0.727-0.772 | 10.464 | ＜0.001 | 0.202 |

Model ^1^: NT-proBNP

Model ^2^: NT-proBNP+age+sex

Model ^3^:

NT-proBNP+age+sex+smoking+drinking+hypertension+diabetes+hyperlipidemia+LVEF+proteinuria+previous MI+PCI or CABG+use of antihypertensives+and use of antilipidemic

**Table S4. Association between the NT-proBNP and the number of carotid artery plaque**

| Number of carotid artery plaque | Variables | OR (95% CI) ^a^ | *P-*value | OR (95% CI) ^b^ | *P-*value | OR (95% CI) ^c^ | *P-*value |
| --- | --- | --- | --- | --- | --- | --- | --- |
| 1 | Continuous  (per 1 SD increase) | 1.38(1.04-1.84) | 0.026 | 1.21(0.94-1.56) | 0.139 | 0.72(0.39-1.33) | 0.288 |
|  | < 56 | Reference |  | Reference |  | Reference |  |
|  | [56-480] | 1.26(0.96-1.65) | 0.092 | 1.03(0.78-1.36) | 0.821 | 1.09(0.77-1.53) | 0.627 |
|  | > 480 | 1.23(0.91-1.67) | 0.185 | 1.06(1.04-1.07) | 0.899 | 1.26(0.81-1.94) | 0.308 |
| ≥2 | Continuous  (per 1 SD increase) | 1.80-1.46-2.22 | <0.001 | 1.40(1.16-1.70) | 0.001 | 1.45(1.06-1.99) | 0.019 |
|  | < 56 | Reference |  | Reference |  | Reference |  |
|  | [56-480] | 1.57(1.33-1.86) | <0.001 | 1.12(0.93-1.34) | 0.244 | 1.02(0.82-1.28) | 0.839 |
|  | > 480 | 2.68(2.22-3.23) | <0.001 | 1.67(1.37-2.05) | <0.001 | 1.74(1.31-2.32) | <0.001 |

^a^ Model 1: unadjusted

^b^ Model 2: adjusted for age, sex

^c^ Model 3: adjusted for age, sex, smoking, drinking, hypertension, diabetes, hyperlipidemia, LVEF, proteinuria, previous MI, PCI or CABG, use of antihypertensives, and use of antilipidemic

**Table S5. Association between the NT-proBNP and the echogenicity of carotid artery plaque**

| Carotid plaque echogenicity | Variables | OR (95 %CI) ^a^ | *P-*value | OR (95 %CI) ^b^ | *P-*value | OR (95 %CI) ^c^ | *P-*value |
| --- | --- | --- | --- | --- | --- | --- | --- |
| Hypoechoic | < 56 | 1.64(1.22-2.21) | 0.001 | 1.51(1.11-2.05) | 0.008 | 1.50(1.02-2.22) | 0.040 |
|  | [56-480] | 0.71(0.51-0.99) | 0.045 | 0.75(0.54-1.04) | 0.086 | 0.68(0.45-1.03) | 0.066 |
|  | > 480 | 0.88(0.59-1.11) | 0.192 | 0.85(0.62-1.18) | 0.343 | 0.96(0.62-1.50) | 0.866 |
| Isoechoic | < 56 | 1.33(1.06-1.68) | 0.015 | 1.18(0.93-1.49) | 0.184 | 0.96(0.72-1.27) | 0.769 |
|  | [56-480] | 0.96(0.76-1.23) | 0.766 | 1.03(0.81-1.31) | 0.812 | 1.02(0.77-1.35) | 0.894 |
|  | > 480 | 0.76(0.60-1.00) | 0.033 | 0.81(0.63-1.05) | 0.112 | 1.03(0.74-1.44) | 0.852 |
| Hyperechoic | < 56 | 0.67(0.60-0.75) | <0.001 | 0.86(0.76-0.97) | 0.014 | 0.89(0.76-1.05) | 0.155 |
|  | [56-480] | 1.03(0.92-1.15) | 0.656 | 0.96(0.85-1.08) | 0.510 | 0.97(0.84-1.14) | 0.736 |
|  | > 480 | 1.45(1.29-1.63) | <0.001 | 1.21(1.07-1.37) | 0.002 | 1.19(1.00-1.42) | 0.050 |
| Mixture | < 56 | 0.51(0.44-0.58) | <0.001 | 0.75(0.64-0.88) | <0.001 | 0.79(0.64-0.97) | 0.019 |
|  | [56-480] | 1.05(0.90-1.21) | 0.561 | 0.93(0.79-1.09) | 0.368 | 0.88(0.72-1.07) | 0.206 |
|  | > 480 | 2.08(1.76-2.45) | <0.001 | 1.54(1.29-1.84) | <0.001 | 1.74(1.35-2.25) | <0.001 |

^a^ Model 1: unadjusted

^b^ Model 2: adjusted for age, sex

^c^ Model 3: adjusted for age, sex, smoking, drinking, hypertension, hyperlipidemia, LVEF, proteinuria, previous MI, PCI or CABG, use of antihypertensives, and use of antilipidemic
